# Priority implementation research for measles-rubella microarray patches identified using the Child Health and Nutrition Research Initiative methodology

**DOI:** 10.7189/jogh.16.04141

**Published:** 2026-04-30

**Authors:** Sara Sá Silva, Melissa Ko, Thomas Cherian, Shamim Qazi, Diana Chang Blanc, Mercy Mvundura, Christopher Morgan, Sonali Kochhar, Auliya Suwantika, Bruce Innis, Katrina Kretsinger, Natasha Crowcroft, Courtney Jarrahian, Anna Thorson, Mahnaz Vahedi, Oniovo Efe-Aluta, Jessica Joyce Mistilis, Birgitte Giersing, Mateusz Hasso-Agopsowicz

**Affiliations:** 1 MMGH Consulting GmbH, Geneva, Switzerland; 2 Independent consultant, Geneva, Switzerland; 3 Independent consultant, Geneva, Switzerland; 4 World Health Organization, Geneva, Switzerland; 5 PATH, Seattle, Washington, USA; 6 JHPIEGO, Baltimore, Maryland, USA; 7 University of Washington, Seattle, Washington, USA; 8 Universitas Padjadjaran, Sumedang, Indonesia; 9 Independent consultant, Haverford, Pennsylvania, USA; 10 Independent consultant, Atlanta, Georgia, USA; 11 Dalla Lana School of Public Health, University of Toronto, Toronto, Ontario, Canada; 12 TDR: the Special Programme for Research and Training in Tropical Diseases, World Health Organization, Geneva, Switzerland

## Abstract

**Background:**

Measles and rubella continue to be a significant global health challenge, disproportionately impacting marginalised communities in low- and middle-income countries (LMICs). Innovative technologies such as measles-rubella microarray patches (MR-MAPs) are currently in development and can potentially improve immunisation coverage and equity through their unique product characteristics. Nonetheless, the limited evidence around their use cases, optimal implementation, cost-effectiveness, and integration into national immunisation programmes raises the need for the development of a prioritised implementation research agenda.

**Methods:**

We used the Child Health and Nutrition Research Initiative (CHNRI) methodology to prioritise 36 research questions (RQs), which we identified through a rapid literature assessment and consultations with experts. We then prepared an online survey and asked stakeholders to assess each question considering the selected CHNRI criteria. We calculated research priority scores (RPS) and average expert agreement (AEA) and conducted stratified analyses restricted to: LMIC government representatives, respondents based in middle-income countries, those with at least moderate experience in implementing health products in LMICs, those with at least moderate knowledge of MR-MAPs, and representatives from academic or research institutions.

**Results:**

A total of 139 respondents identified a diverse range of priorities for MR-MAP implementation research. The 36 RQs had a median RPS of 80%. The key priorities identified included research on vaccine supply chain management, vaccine uptake among underserved populations, and human resource implications for integration in routine immunisation services. Stratified analyses revealed a diversity of scores, with representatives from LMIC governments generally prioritising operational questions related to human resources, vaccine administration, and managing dual delivery mechanisms. Fifteen RQs were prioritised to accommodate different perspectives.

**Conclusion:**

This exercise prioritised implementation questions that will inform MR-MAPs’ research investments and efforts over the next 10 years in order to prepare for their introduction in LMICs. While the questions identified in this exercise were specifically about MR-MAPs, the RQs and potential evidence may apply to other vaccine MAPs. This research addresses critical evidence needed towards the successful roll-out of this innovative technology in LMICs, to ensure equitable access to measles and rubella vaccination and accelerate progress towards measles and rubella control and elimination.

Measles and rubella remain significant public health challenges, with an estimated 107 500 measles deaths in 2023, and ~ 32 000 infants born with congenital rubella syndrome each year [1,2]. These challenges are particularly acute in low- and middle-income countries (LMICs), where resource-constrained immunisation systems face barriers to reaching underserved populations. To achieve elimination, the World Health Organization (WHO) recommends that countries obtain and sustain a coverage 95% with two doses of measles-containing vaccine (MCV) and that a rubella-containing vaccine be provided in combination with MCV [3,4]. Despite global efforts to increase measles coverage, progress remains fragile. According to WHO/United Nations Children’s Fund (UNICEF) estimates for national immunisation coverage for 2024, global MCV1 coverage was 84% in 2024, still below the 2019 level of 86%, leaving 1.4 million more children unprotected compared to before the COVID-19 pandemic (*i.e.* 2019). Global MCV2 coverage reached 75% in 2024, an improvement from 71% in 2019, but remains insufficient to close immunity gaps [5]. These persistent gaps highlight the urgent need for innovations that can strengthen delivery, reduce missed opportunities, and expand access to hard-to-reach populations. The stagnation in coverage highlights the need for innovative solutions to enhance equitable coverage in measles and rubella-containing (MR) vaccines.

Measles-rubella microarray patches (MR-MAPs) are innovative devices designed to address persistent challenges in MR vaccine delivery, including cold chain dependence, high open-vial wastage, the need for highly trained health workers, and risk of administration errors [6,7]. They are a single dose, ready-to-use presentation with enhanced thermostability that eliminates the need for reconstitution and does not require subcutaneous injection [6-9]. MR-MAPs have completed phase 2 development and are anticipated to become available by 2030. By simplifying administration, they could reduce burden on skilled vaccinators and expand the workforce ultimately improving access to vaccines. Their single-dose format eliminates open vial wastage, which is currently high, as vials must be discarded six hours after reconstitution. Moreover, MR-MAPs could be used in controlled temperature chain conditions, facilitating the reach of un- and under-immunised children in settings where cold chain infrastructure is limited [10].

When MR-MAPs are licenced, their initial supply is anticipated to be constrained due to limited manufacturing and a higher price compared to the needle and syringe presentation, which is in multi-dose vials. Thus, several questions remain around their large-scale implementation in LMICs, such as their acceptability, affordability, sustainable integration into national immunisation programmes, and their ability to reach underserved populations [6]. As MR-MAPs advance in clinical development, implementation research is needed to generate evidence on their use in real-world conditions and address the operational and contextual challenges to ultimately better inform developers and decision-makers and decrease delays from authorisation to implementation in LMICs.

To guide the introduction and scale-up of MR-MAPs in LMICs, we developed priority implementation research questions (RQs) for MR-MAPs using the Child Health and Nutrition Research Initiative (CHRNI) methodology [11,12]. This methodology is widely used in global health and provides an evidence-based way to set priorities in resource-limited settings. While it has several advantages, such as its systematic, transparent, flexible, and low-cost process, we largely selected it for its systematic and inclusive approach that engages a wide range of stakeholders at the national, regional, and global levels to build consensus, ownership, and legitimacy of the results [13,14]. Its structured scoring system of RQs reduces individual bias and prevents dominance using predefined criteria.

## METHODS

The implementation research prioritisation process was conducted in two phases.

### Phase 1

The CHNRI method requires defining and agreeing on the research context and criteria that determine the prioritisation of RQs. Thus, the first phase focused on adapting a common method for research priority setting developed by the CHNRI to the MR vaccine context and establishing the criteria for scoring the implementation RQs [11].

The project team drafted the context and criteria, which were then vetted through discussions at the WHO Global Convening on Measles and Rubella Monitoring and Assessment Platforms (MR-MAPs) in April 2024, held in New Delhi, India. This gathering included experts in measles and rubella, regulators, policymakers, developers of MAPs, and vaccine manufacturers. The criteria were subsequently recommended by the WHO Technical Advisory Group (TAG) on MR-MAPs [15]. The criteria were selected to meet the goals of the implementation research priorities focused on generating data and evidence on how MR-MAPs can be used within real-world conditions (Table S1 in the **Online Supplementary Document**).

**Figure 1:**
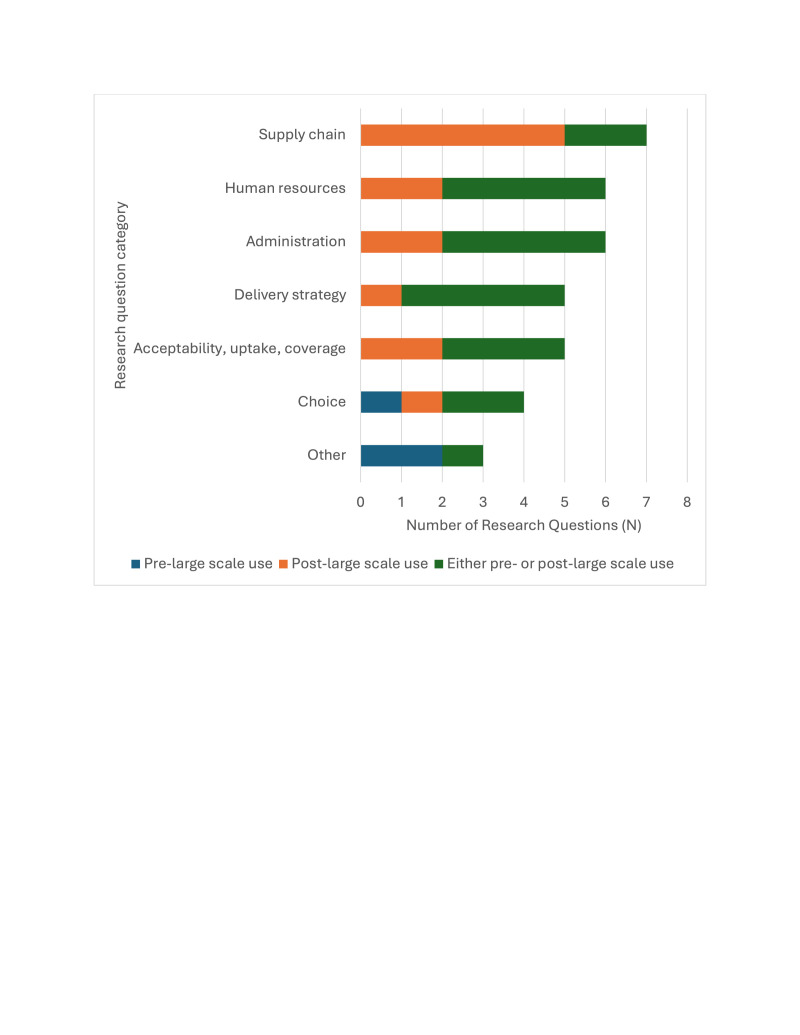
Number of research questions per category.

Evidence needs related to MR-MAP implementation were identified through two main sources: a desk review of published and unpublished literature, and break-out group discussions at the Global Convening on MR-MAPs. Through this method, we identified 68 evidence needs that were converted into 47 draft RQs, after removing duplications and grouping evidence needs that could be consolidated into one RQ. The 47 draft RQs underwent an iterative review process where 10 stakeholders (global MR experts, WHO MR regional focal points, members of the MR-MAP TAG, vaccine implementation experts, and implementation research experts) provided their comments. This resulted in the further removal of duplications and redundancies, standardisation of the language, and revisions to wording to improve clarity. We categorised the final list of 36 RQs selected for prioritisation into seven groups using the WHO implementation research guidance as a reference [16]. Following feedback from the MR-MAP TAG, the RQs were also categorised to provide indicative timing of when the research should take place to be most impactful for implementation considering three potential categories: pre-large-scale use, post large-scale use, or either pre- or post- large-scale use (**Figure 1**).

### Phase 2

This phase used the CHNRI methodology to prioritise the identified RQs [11]. We designed an online survey using Qualtrics, version August 2024 (Qualtrics International Inc., Provo, Utah, USA) for respondents to score the 36 RQs using the defined context and criteria (Table S2 in the **Online Supplementary Document**). The survey first asked four demographic questions on a respondent’s geographical location, organisational affiliation, familiarity with MR-MAPs, and the level of experience implementing health interventions in LMICs. Then, the 36 RQs were organised into groups by research topic. The categories of questions were randomised for each survey participant to allow for the inclusion of partial responses without bias towards one category of questions and to reduce potential biases created due to survey fatigue. The respondents were given options of ‘Yes’, ‘No’, and ‘Partially’ for questions that they felt they could answer, which were assigned 1, 0, and 0.5 points, respectively. They could also answer ‘don’t know’ for those questions that they felt were outside their area of expertise and knowledge; such responses were excluded from the analysis when calculating research priority scores (RPSs).

An anonymous link to complete the Qualtrics survey was sent to 165 individuals identified by the project team based on their expertise in measles and rubella vaccines and immunisation programme implementation in LMICs. Respondents included WHO national, regional, and global MR focal points, programme managers, and immunisation officers and the global MR-MAP Technical Advisory Group. Participation in the survey was voluntary. As the survey was accessed *via* an anonymous link, it was further shared by the original 165 individuals and the project team to other relevant stakeholders in efforts to increase response rates from individuals with experience in LMICs. The online survey remained open for approximately two months, and regular reminders were sent every two weeks to invitees. One of the 36 RQs was inadvertently excluded in the original survey and was subsequently scored through a separate Qualtrics survey distributed to the above 165 individuals.

All responses were downloaded from Qualtrics into Microsoft Excel, version 2024 (Microsoft Corporation, Redmond, Washington, USA). Partially completed surveys where at least one response was provided to score a RQ were included in the analysis. Then, the RPS and average expert agreement (AEA) were calculated for each RQ.

The RPS is a composite score that reflects the collective priority of a research option, calculated as the average of scores assigned by experts across the predefined criteria. The following formula was used to calculate the RPS, where ‘c’ is the five criteria evaluating the RQ.



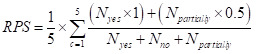



We calculated 95% confidence intervals (CIs) using respondent-level data to assess the uncertainty of each RPS. Specifically, we divided the standard deviation of respondent-level scores by the square root of the number of respondents to obtain the standard error. The 95% CI was then calculated as:



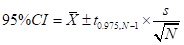



Here, *x̄* is the mean respondent-level score, and *t*_(0.975,_*_N _*_− 1)_ is the critical value from the respondent’s *t* distribution with *N *− 1degrees of freedom. In cases where all respondents provided identical scores (zero variance), the CI collapsed to the point estimate (*e.g.* 0.80–0.80); this was reported as ‘not estimable’ in tables, indicating no measurable variability. The 95% CIs were truncated to the 0–100 range to reflect the bounds of possible scores.

Meanwhile, AEA measures the level of consensus among experts by calculating the proportion of pairwise agreements in their scoring across all evaluation criteria. It was calculated using the following formula.



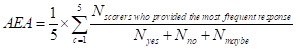



Finally, we calculated stratified average RPS and AEA scores to identify any differences in scores based on the respondents’ organisational affiliations, geographical location, and level of experience in implementing health products and interventions in LMICs.

## RESULTS

A total of 139 individuals participated in scoring the RQs, with 95 (68%) fully completing the survey and 44 (32%) providing partial responses (Table S2 in the **Online Supplementary Document**). We received 34 responses for the RQ that was scored separately. Despite additional efforts to increase response rates from LMIC government representatives, this group remained relatively small (n = 14). Most respondents (n = 103) reported at least moderate familiarity with MR-MAPs.

When all survey responses were aggregated for all respondents, the RPS ranged from 70–93%, suggesting that between 70% and 93% of the respondents considered the questions as highly important, with a median of 80% indicating a strong consensus around the importance of most RQs. The AEA ranged from 53–87%, indicating moderate to high agreement, with higher agreement scores for RQs with higher RPS. This suggests that, when an RQ was recognised as important (high RPS), respondents tended to agree on its prioritisation (high AEA). We evaluated the impact of weighting the criteria and found that it did not substantially impact the prioritisation results unless a single criterion was weighted disproportionately (>50%) higher than others. We consequently applied no weighting to the criteria.

Besides the analysis for all respondents, we also performed five stratified analyses considering only responses from individuals that represented LMIC governments (n = 14), were located in middle-income countries (n = 78), indicated at least moderate experience in implementing health products and interventions in LMICs (n = 116), indicated at least moderate knowledge of MR-MAPs (n = 103), and were affiliated with academic or research institutions (n = 27) (**Table 1**). The stratified analysis showed that those with at least moderate experience in implementing health products or interventions in LMICs and those with at least moderate knowledge of MR-MAPs had similar priorities for the top 10 highest-scoring RQs. Similarly, the stakeholders from academic or research institutions had similar priorities, but they prioritised RQs related to the supply chain, particularly the impact of the controlled temperature chain and the use of MR-MAPs in older ages and self-administration. In contrast, the analysis with only respondents that represented LMIC governments showed significantly different scores compared to all respondents. (**Table 2**).

**Table 1:** Overview of RPS by respondent demographics

		% of RPS					
**Research question**	**Survey category**	**All responders (n = 139)**	**Government in LMICs (n = 14)**	**Located in LMICs (n = 78)**	**Experience in LMICs (n = 116)**	**MR-MAP knowledge (n = 103)**	**Academic and research institutes (n = 27)**
What would be the impact on cold chain management and logistics of having thermostable MR-MAPs (*e.g.* vaccine vial monitors with high stability of 30 d at +37°C or the ability to tolerate ambient temperatures of at least +40°C for a minimum of three days)?*	Supply chain	93	80	93	93	92	91
Can the use of MR-MAPS reduce MOVs, improve compliance in people with needle phobia, or reach children in hard-to-vaccinate communities? If so, by how much and what is the estimated impact on overall coverage?	Acceptability, uptake, and coverage	89	88	91	87	88	98
How can MR-MAPs improve MR vaccine uptake by and access to un- and under-vaccinated children, and to what extent, compared to the injectable vaccine?	Acceptability, uptake, and coverage	87	87	90	86	87	95
What conditions and criteria need to be met to enable the optimal use (*e.g.* increase coverage while minimising wastage) of MR-MAPs under controlled temperature conditions or the ability to tolerate ambient temperatures of at least +40°C for a minimum of three days?	Supply chain	87	95	88	87	87	90
What is the impact of MR-MAPs on human resources (e.g. delivery strategies, team numbers, team composition, use of lesser trained personnel) planning for routine and SIAs?	Human Resources	87	90	90	86	87	87
What would be the role of MR-MAPs be in facilitating a timely response to measles or rubella outbreaks?	Delivery strategy	85	90	90	85	85	82
What would be the incremental benefit on vaccine coverage of having thermostable MR-MAPs (*e.g.* ability to tolerate ambient temperature of +40°C for a minimum of three days or vaccine vial monitors with high stability of 30 d at +37°C)?	Supply chain	85	86	86	85	86	94
What are the caregiver and vaccinator perceptions on the risks, challenges, and opportunities of co-administration of MR-MAPs with other vaccines and/or with other health interventions (*e.g.* Vitamin A, deworming)?	Administration	85	92	88	85	83	88
What are the caregiver and vaccinator perceptions on the risks, challenges, and opportunities of MR-MAPs that could affect acceptability and/or uptake?	Acceptability, uptake, and coverage	84	93	87	83	84	89
Can MR-MAPs reduce refusals related to multiple injections in one visit; if so, what is the frequency?	Acceptability, uptake, and coverage	83	89	88	81	82	86
What are the drivers of costs of MR-MAP in different delivery strategies and contexts relative to the full benefits such as increase in coverage and reduction in measles cases compared with injectable MR vaccines?	Delivery strategy	83	89	86	82	81	84
What are the incremental benefits of different approaches or use cases (such as use in specific populations, use in specific delivery strategies) for the roll-out for MR-MAPs?	Delivery strategy	82	93	87	83	82	80
What are the caregiver and vaccinator perceptions about MR-MAPs being kept out of the cold chain if controlled temperature chain conditions (*e.g.* the ability to tolerate ambient temperatures of at least +40°C for a minimum of three days) are used?	Supply chain	82	86	88	83	82	89
What are the caregiver and vaccinator perceptions on the choice between MR-MAPs and MR injectable presentation when both are available?	Choice	82	91	87	82	82	85
What are the feasible delivery strategies to reach older age groups (*e.g.* school-aged, adolescents, young adults, etc.) with MR-MAPs?	Delivery strategy	82	94	84	80	78	89
What is the impact of the anticipated shelf life (*e.g.* 24 mo or greater) of MR-MAPs on procurement, distribution and storage?	Supply chain	81	89	88	80	79	90
What are the factors that would influence a national government's choice between MR-MAPs and MR five-dose vials in national immunisation programmes for different use cases?	Choice	80	83	84	80	80	82
What are the effective strategies, approaches, and tools to influence community behaviour and practices and increase the acceptance and/or uptake of MR-MAPs at national and sub-national levels among various stakeholders?	Acceptability, uptake, and coverage	80	76	81	78	78	85
What are the training strategies and tools both initial and refresher for different categories of vaccinators (including community health workers or volunteers) for MR-MAP implementation?	Human resources	80	96	85	79	79	87
What measures are required to adequately prepare community health worker and/or volunteers (*i.e.* lesser trained) to administer MR-MAPs and identify, respond, and report adverse events?	Human resources	80	78	84	81	82	84
What are the learnings from community health worker/community health volunteers’ administration from other vaccines (*e.g.* oral polio vaccine) and novel delivery devices such as compact pre-filled auto disabled injection devices used in community settings that may apply to MR-MAPs?	Other	79	98	83	79	79	80
What is the feasibility of self-administration (*e.g.* includes caregiver administration) of MR-MAPs?	Administration	79	79	79	80	80	88
What are the frequency and the causes of MR-MAP administration errors (including errors in wear time) and their programmatic implications?	Administration	79	88	83	80	79	84
What are the risks, challenges, and opportunities for targeting the use of MR-MAPs (*e.g.* only geographically or for specific populations) while using injectable vaccines for the rest of the population?	Delivery strategy	79	83	78	78	78	85
Is the site of administration of MR-MAPs likely to influence the acceptability and/or uptake?	Administration	79	93	82	80	79	83
What are the factors that impact a national immunisation programme's willingness to pay a higher price for MR-MAPs in return for its potential benefits?	Other	79	74	82	77	78	83
Would the use of MR-MAPs under controlled temperature chain conditions (*e.g.* the ability to tolerate ambient temperatures of at least +40°C for a minimum of three days) in outreach/campaign activities increase the wastage of unused vaccines compared to use under standard cold chain (*e.g.* as vaccines under controlled temperature chain conditions cannot be restored in the refrigerators, would MR-MAPs be reallocated to other activities to avoid closed vial or unused vaccine wastage)?	Supply chain	78	81	84	77	76	85
What are the waste management and planning needs for MR-MAPs considering different delivery strategies in comparison to other vaccines, including the MR injectable vaccine?	Supply chain	77	82	83	76	75	83
What are the factors impacting successful management and use of different vaccine presentations of MR, considering national policymaker, national and subnational programme managers, and vaccinator perspectives?	Choice	77	94	85	78	74	74
What are the factors that are likely to influence MR-MAP wear time when administered by community health worker/volunteer during house-to-house activities?	Human resources	77	95	84	77	76	79
What are the most appropriate supervision and monitoring practices of vaccination sessions to ensure the effective implementation of MR-MAPs?	Human resources	77	91	82	77	75	82
What are the key drivers that a vaccinator would consider when choosing to deliver MR-MAPs or MR injectable vaccine when both are available? What is the frequency and rationale of the vaccinator for opting for one presentation over the other?	Choice	76	93	83	75	72	83
Can caregivers ensure children meet the required wear time after the administration of the MR-MAP?	Human resources	75	97	81	75	74	81
How does the administration of MR-MAPs influence the quality-of-service delivery?	Administration	74	96	88	73	73	84
What are the environmental factors and their frequency that can affect the appropriate use of MR-MAPs?	Administration	73	90	84	74	71	80
What are the learnings from a novel delivery device such as needle-free jet injectors, compact pre-filled auto disabled injection devices, and other presentations that can inform the introduction and use of MR-MAPs?	Other	70	93	76	68	67	72

LMIC – low- and middle-income country, MOV – missed opportunities for vaccination, MR – measles-rubella, MR-MAP – measles-rubella microarray patch, RPS – research priority score, RQ – research question, SIA – supplementary immunisation activity

*This research question was scored separately from the other research questions.

**Table 2:** Ranking of the 36 RQs by survey category across all respondents (n = 139) and by government officials in LMICs (n = 14) based on RPSs

		All respondents (n = 139)			Governments in LMICs (n = 14)		
**RQ**	**Timing of research**	**% of RPS (95% CI)**	**% of AEA**	**Ranking**	**% of RPS (95% CI)**	**% of AEA**	**Ranking**
Acceptability, uptake, and coverage							
*Can the use of MR-MAPS reduce MOVs, improve compliance in people with needle phobia, or reach children in hard-to-vaccinate communities? If so, by how much and what is the estimated impact on overall coverage?*	Post	89 (83–95)	80	2	88 (61–100)	76	23
*How can MR-MAPs improve MR vaccine uptake by and access to un- and under-vaccinated children, and to what extent, compared to the injectable vaccine?*	Post	87 (82–93)	77	3	87 (60–100)	75	25
*What are the caregiver and vaccinator perceptions on the risks, challenges, and opportunities of MR-MAPs that could affect acceptability and/or uptake?*	Pre or post	84 (78–90)	71	9	93 (66–100)	86	13
*Can MR-MAPs reduce refusals related to multiple injections in one visit; if so, what is the frequency?*	Pre or post	83 (76–89)	72	10	89 (62–100)	78	20
*What are the effective strategies, approaches, and tools to influence community behaviour and practices and increase the acceptance and/or uptake of MR-MAPs at national and sub-national levels among various stakeholders?*	Pre or post	80 (74–87)	64	18	76 (49–100)	53	34
Administration							
*What are the caregiver and vaccinator perceptions on the risks, challenges, and opportunities of co-administration of MR-MAPs with other vaccines and/or with other health interventions (e.g. vitamin A, deworming)?*	Pre or post	85 (79–90)	73	8	92 (71–100)	86	14
*What is the feasibility of self-administration (e.g. includes caregiver administration) of MR-MAPs?*	Pre or post	79 (72–86)	68	22	79 (50–100)	74	32
*What are the frequency and the causes of MR-MAP administration errors (including errors in wear time) and their programmatic implications?*	post	79 (73–85)	64	23	88 (59–100)	75	24
*Is the site of administration of MR-MAPs likely to influence the acceptability and/or uptake?*	Pre or post	79 (72–85)	67	25	93 (71–100)	86	12
*How does the administration of MR-MAPs influence the quality-of-service delivery?*	Post	74 (67–81)	57	34	96 (65–100)	93	3
*What are the environmental factors and their frequency that can affect the appropriate use of MR-MAPs?*	Pre or post	73 (66–81)	57	35	90 (60–100)	80	19
Delivery strategy							
*What would be the role of MR-MAPs be in facilitating a timely response to measles or rubella outbreaks?*	Pre or post	85 (79–92)	75	6	90 (57–100)	83	18
*What are the drivers of costs of MR-MAP in different delivery strategies, and contexts relative to the full benefits such as increase in coverage and reduction in measles cases compared with injectable MR vaccines?*	Pre or post	83 (77–89)	70	11	89 (61–100)	82	22
*What are the incremental benefits of different approaches or use cases (such as use in specific populations, use in specific delivery strategies) for the roll-out for MR-MAPs?*	Post	82 (76–89)	69	12	93 (67–100)	89	11
*What are the feasible delivery strategies to reach older age groups (e.g. school-aged, adolescents, young adults, etc.) with MR-MAPs?*	Pre or post	82 (76–88)	69	15	94 (68–100)	91	7
*What are the risks, challenges, and opportunities for targeting the use of MR-MAPs (e.g. only geographically or for specific populations) while using injectable vaccines for the rest of the population?*	Pre or post	79 (72–85)	63	24	83 (57–100)	69	28
Human resources							
*What is the impact of MR-MAPs on human resources (e.g. delivery strategies, team numbers, team composition, use of lesser trained personnel) planning for routine and SIAs?*	Post	87 (81–93)	75	5	90 (60–100)	80	17
*What are the training strategies and tools both initial and refresher for different categories of vaccinators (including community health workers or volunteers) for MR-MAP implementation?*	Pre or post	80 (74–87)	68	19	96 (67–100)	91	4
*What measures are required to adequately prepare community health worker and/or volunteers (i.e. lesser trained) to administer MR-MAPs and identify, respond, and report adverse events?*	Pre or post	80 (73–87)	66	20	78 (48–100)	69	33
*What are the factors that are likely to influence MR-MAP wear time when administered by community health worker / volunteer during house-to-house activities?*	Pre or post	77 (70–83)	62	30	95 (65–100)	90	6
*What are the most appropriate supervision and monitoring practices of vaccination sessions to ensure the effective implementation of MR-MAPs?*	post	77 (70–83)	59	31	91 (64–100)	82	15
*Can caregivers ensure children meet the required wear time after the administration of the MR-MAP?*	Pre or post	75 (68–82)	59	33	97 (64–100)	94	2
Other							
*What are the learnings from community health worker/community health volunteers’ administration from other vaccines (e.g. oral polio vaccine) and novel delivery devices such as compact pre-filled auto disabled injection devices used in community settings that may apply to MR-MAPs?*	Pre	79 (73–86)	65	21	98 (71–100)	96	1
*What are the factors that impact a national immunisation programme's willingness to pay a higher price for MR-MAPs in return for its potential benefits?*	Pre or post	79 (72–85)	65	26	74 (43–100)	67	35
*What are the learnings from a novel delivery device such as needle-free jet injectors, compact pre-filled auto disabled injection devices, and other presentations that can inform the introduction and use of MR-MAPs?*	Pre	70 (63–77)	53	36	93 (65–100)	89	10
Product choice							
*What are the caregiver and vaccinator perceptions on the choice between MR-MAPs and MR injectable presentation when both are available?*	Pre or post	82 (76–88)	69	14	91 (64–100)	84	16
*What are the factors that would influence a national government's choice between MR-MAPs and MR five-dose vials in national immunisation programmes for different use cases?*	Pre	80 (74–87)	66	17	83 (55–100)	75	29
*What are the factors impacting successful management and use of different vaccine presentations of MR, considering national policymaker, national and subnational programme managers, and vaccinator perspectives?*	Post	77 (70–83)	58	29	94 (67–100)	89	8
*What are the key drivers that a vaccinator would consider when choosing to deliver MR-MAPs or MR injectable vaccine when both are available? What is the frequency and rationale of the vaccinator for opting for one presentation over the other?*	Pre or post	76 (69–82)	59	32	93 (66–100)	89	9
Supply chain							
What would be the impact on cold chain management and logistics of having thermostable MR-MAPs (e.g. vaccine vial monitors with high stability of 30 d at +37°C or the ability to tolerate ambient temperatures of at least +40°C for a minimum of three days)?*	Post	93 (85–99)	87	1	80 (not estimable)	100	23
*What conditions and criteria need to be met to enable the optimal use (e.g. increase coverage while minimising wastage) of MR-MAPs under controlled temperature conditions or the ability to tolerate ambient temperatures of at least +40°C for a minimum of three days?*	Post	87 (83–91)	76	4	95 (88–100)	91	5
*What would be the incremental benefit on vaccine coverage of having thermostable MR-MAPs (e.g. ability to tolerate ambient temperatures of +40°C for a minimum of three days or vaccine vial monitors with high stability of 30 d at +37°C)?*	Post	85 (81–90)	75	7	86 (66–100)	82	26
*What are the caregiver and vaccinator perceptions about MR-MAPs being kept out of the cold chain if controlled temperature chain conditions (e.g. the ability to tolerate ambient temperatures of at least +40°C for a minimum of three days) are used?*	Pre or post	82 (78–87)	71	13	86 (72–100)	75	27
*What is the impact of the anticipated shelf life (e.g. 24 mo or greater) of MR-MAPs on procurement, distribution and storage?*	Post	81 (75–87)	70	16	89 (76–100)	81	21
*Would the use of MR-MAPs under controlled temperature chain conditions (e.g. the ability to tolerate ambient temperatures of at least +40°C for a minimum of three days) in outreach/campaign activities increase the wastage of unused vaccines compared to use under standard cold chain (e.g. as vaccines under controlled temperature chain conditions cannot be restored in the refrigerators, would MR-MAPs be reallocated to other activities to avoid closed vial or unused vaccine wastage)?*	Post	78 (71–85)	64	27	81 (59–100)	73	31
*What are the waste management and planning needs for MR-MAPs considering different delivery strategies in comparison to other vaccines, including the MR injectable vaccine?*	Pre or post	77 (71–83)	63	28	82 (62–100)	72	30

AEA – average expert agreement, CI – confidence interval, LMIC – low- and middle-income country, MOV – missed opportunities for vaccination, MR – measles-rubella, MR-MAP – measles-rubella microarray patch, RPS – research priority score, RQ – research question, SIA – supplementary immunisation activity

*This research question was scored separately from the other research questions.

Various options regarding the selection of which prioritised RQs were considered and ultimately a blended approach was suggested considering the top 10 ranked RQs from all respondents and the top 5 ranked RQs from government representatives in LMICs that are not included in the top 10. Using this criterion, we identified the list of 15 prioritised RQs (**Table 3**). This consolidation was done since the ranking by the government representatives differed from those of the others, but were considered important because they addressed issues that are considered essential for successful implementation at the national level. This approach did not drop any high-scoring RQs from all respondents, while also allowing us balance different perspectives and to highlight the key priorities from government stakeholders who will ultimately be responsible for adopting, implementing, and financing MR-MAP.

**Table 3:** Top 15 prioritised RQs for the implementation research agenda for MR-MAP

RQ	Ranking	
**All**	**LMIC governments**
What would be the impact on cold chain management and logistics of having thermostable MR-MAPs (*e.g.* vaccine vial monitors with high stability of 30 d at +37°C or the ability to tolerate ambient temperatures of at least +40°C for a minimum of three days)?*	1	23
Can the use of MR-MAPS reduce MOVS, improve compliance in people with needle phobia, or reach children in hard-to-vaccinate communities? If so, by how much and what is the estimated impact on overall coverage?	2	23
How can MR-MAPs improve MR vaccine uptake by and access to un- and under-vaccinated children, and to what extent, compared to the injectable vaccine?	3	25
What conditions and criteria need to be met to enable the optimal use (*e.g.* increase coverage while minimising wastage) of MR-MAPs under controlled temperature conditions or the ability to tolerate ambient temperatures of at least +40°C for a minimum of three days?	4	5
What is the impact of MR-MAPs on human resources (*e.g.* delivery strategies, team numbers, team composition, use of lesser trained personnel) planning for routine and SIAs?	5	17
What would be the role of MR-MAPs be in facilitating a timely response to measles or rubella outbreaks?	6	18
What would be the incremental benefit on vaccine coverage of having thermostable MR-MAPs (*e.g.* ability to tolerate ambient temperatures of +40°C for a minimum of three days or vaccine vial monitors with high stability of 30 d at +37°C)?	7	26
What are the caregiver and vaccinator perceptions on the risks, challenges, and opportunities of co-administration of MR-MAPs with other vaccines and/or with other health interventions (*e.g.* Vitamin A, deworming)?	8	14
What are the caregiver and vaccinator perceptions on the risks, challenges, and opportunities of MR-MAPs that could affect acceptability and/or uptake?	9	13
Can MR-MAPs reduce refusals related to multiple injections in one visit, if so, what is the frequency?	10	20
What are the learnings from community health worker/community health volunteers’ administration from other vaccines (*e.g.* oral polio vaccine) and novel delivery devices such as compact pre-filled auto disabled injection devices used in community settings that may apply to MR-MAPs?	21	1
Can caregivers ensure children meet the required wear time after the administration of the MR-MAP?	33	2
How does the administration of MR-MAPs influence the quality of service delivery?	34	3
What are the training strategies and tools both initial and refresher for different categories of vaccinators (including community health workers or volunteers) for MR-MAP implementation?	19	4
What are the factors that are likely to influence MR-MAP wear time when administered by community health worker / volunteer during house-to-house activities?	30	6

LMIC – low- and middle-income country, MOV – missed opportunities for vaccination, MR-MAP – measles-rubella microarray patch, SIA – supplementary immunisation activity

*This research question was scored separately from the other research questions.

## DISCUSSION

We used the CHNRI methodology to develop and prioritise a set of implementation RQs for MR-MAPs. This standardised, consultative approach allows for wide consultation and engagement of all types of stakeholders in identifying key research priorities. We identified 15 priority RQs out of the 36 evaluated. These RQs demonstrated high RPSs, reflecting strong consensus on their importance among respondents and the diversity of priorities among a range of stakeholders, including representatives from countries that are most likely to benefit from MR-MAPs implementation. Our findings can guide research funding agencies facing investment decisions for implementation research on MR-MAPs and ensure that the most critical evidence needs are being addressed. They can also guide programme planners in formative and summative evaluations of the deployment of MR-MAPs.

Our analysis also revealed an inverse relationship between RPS and AEA, suggesting that the lower scores could indicate expert disagreement on the RQ’s priority rather than consensus on their lower importance. Thus, these results prioritise the RQs that received the highest level of AEA and are clearly important to the stakeholders who responded to the surveys.

The striking finding is the divergence in the priorities between respondents representing the LMIC governments and the broader pool of respondents: the former prioritised operational and workforce concerns tied to programme delivery, while the latter placed greater importance on RQs relating to MR-MAPs acceptability, coverage, and uptake and supply chain RQs. These differences likely reflect individuals’ specific perspectives and experiences, but may also signal disconnections between those working on technology development and those implementing it. If not addressed, MR-MAPs risk being perceived as a top-down technical fix, rather than tools that respond to on-the-ground realities.

The stratified analyses highlight the importance of recognising different perspectives and ensuring that all evidence needs across stakeholder groups are addressed to minimise MR-MAPs implementation. The emphasis on programmatic and workforce considerations by LMIC stakeholders showcases some of the key challenges in integrating MR-MAPs into a resource-constrained immunisation programme and health system and reinforces the need to ensure that local priorities are represented. These findings resonate with broader health systems research on vaccine innovation uptake, which underscores the importance of system readiness, financing, and integration into existing service delivery. Lessons from past innovations, such as nasal spray influenza vaccines and compact pre-filled auto disable (compact pre-filled auto-disable devices or cPAD) devices, demonstrate that technical promise alone does not ensure adoption [17]. MR-MAP implementation research must be grounded in these realities to ensure sustainability and equity.

While 103 respondents indicated at least moderate familiarity, absence of hands-on experience with MR-MAPs may have influenced how confidently they were able to score certain RQs. This reinforces the need for ongoing evidence generation and iterative priority setting as the product moves closer to licensure.

The strengths of this exercise included the consultative process to develop the RQs, which allowed us to consider the different perspectives of those involved in MAP development and the implementation of MR vaccine programmes. We also successfully received a high number of respondents to score the RQs, with a reasonable geographic distribution. Finally, over half of the respondents indicated at least moderate experience with implementing health interventions in LMICs.

Despite these contributions towards successful MR-MAP implementation, this study has some limitations. The inadvertent exclusion of one RQ in the original survey and its subsequent inclusion through a separate survey likely introduced a bias related to that RQ on cold chain management. The smaller respondent pool for this question (only 34 individuals scored this one question compared with ~100 individuals that scored the other 35 questions) and its higher RPS and AEA scores reduce its comparability with the other RQs. Opportunities should be explored to manage the potential misallocation of resources, and this RQ should be combined with others in a single study. Furthermore, the sampling frame initially targeted people known to the MR field with the extension to others *via* the anonymous link. We did not conduct a systematic sample of any group with a defined denominator and response rate. This may have resulted in the low response rates of those individuals who represented LMIC governments who will be essential stakeholders in ensuring appropriate implementation of MR-MAPs. However, the CHNRI evaluation showed that expert opinions on research ideas stabilise quickly, and as few as 45–55 experts are sufficient to reliably reproduce the identified priorities [18].

Further, as this is a global research agenda, we felt that technical knowledge of the vaccine and a reasonable level of understanding of the platform used (*i.e.* MR-MAPs) was required to respond to the survey and thus did not target any community members or marginalised populations. Expanding the engagement of both LMIC representatives and community participants in future discussions can further refine the implementation research agenda to understand their priorities to better mitigate the risk of potential delays in MR-MAP implementation and uptake in LMICs [19]. Opportunities for improvement that should be explored include broadening the scope of stakeholder engagement to ensure a more representative sample, particularly from countries that might benefit the most from implementing MR-MAPs. This could be managed by more qualitative methods (*e.g.* semi-structured surveys, focus groups, or interviews) to obtain richer information from this key group of stakeholders. Finally, as researchers undertake the study design and protocol development, exploration to combine RQs into fewer research projects could also be considered to optimise resources.

Beyond study-specific limitations, there are inherent limitations of the CHNRI methodology. Because the process depends on a set of criteria without allowing for further exploration or qualitative explanation, complex perspectives can be oversimplified [13,20]. Oversimplification can be particularly acute for novel products, such as MR-MAPs, where direct familiarity is limited. Our exercise could be further strengthened by exploring a hybrid approach (*e.g.* combining it with Delphi-style exercise) to further unpack the rationale and prioritisation of certain RQs. Although the high AEA for the high priority RQs suggest consistency, the limitations in CHNRI methodology should be borne in mind. Finally, CHNRI produces a one-time priority list, which may need to be revisited as evidence, policy priorities, and product development evolves.

## CONCLUSIONS

The prioritised RQs have been shared with the MR-MAP TAG and are informing ongoing funding initiatives to address critical evidence gaps. Our CHNRI analysis provides a structured agenda to guide investments in implementation research, ensuring evidence is aligned with the needs of policymakers and implementers. By addressing the identified challenges, stakeholders at global, regional, and national levels can lay the groundwork for equitable and effective MR-MAP introduction. As licensure approaches, this agenda offers a practical roadmap to support donor alignment and timely decision-making for MR-MAP implementation. Given the methodological limitations of CHNRI and the evolving evidence base, this agenda should be viewed as a living resource that will require periodic updating as MR-MAPs move through development and introduction.

## Additional material


Online Supplementary Document
