# Supplementary material for: Priority implementation research for measles-rubella microarray patches identified using the Child Health and Nutrition Research Initiative methodology
Source: J Glob Health. 2026 Apr 30;16:04141. doi: 10.7189/jogh.16.04141 (PMC13132005; doi:10.7189/jogh.16.04141)
Supplement: Online Supplementary Document [file jogh-16-04141-s001.pdf]

Supplement to: Sá Silva S, Ko M, Cherian T, Qazi S, Chang Blanc D, Mvundura M, Morgan C, Kochhar S, Suwantika A, Innis B, Kretsinger K, Crowcroft N, Jarrahian C, Thorson A, Vahedi M, Efe-Aluta O, Mistilis JJ, Giersing B, Hasso-Agopsowicz M. Priority implementation research for measles-rubella microarray patches identified using the Child Health and Nutrition Research Initiative methodology. J Glob Health. 2026;16:04041.

Table S1. Adapted CHNRI context and criteria for measles rubella microarray patch implementation research

**Context**	**Description**
Who (population of interest)	All communities in countries that utilise measles-rubella vaccines in their routine immunisation or for supplementary immunisation activities (preventive or outbreak response). Potential to include special populations in high-income countries (*e.g.* migrant populations, difficult to reach, *etc.*).
Where (geographical scope of research)	Global, regional, country, and sub-national levels.
When (time scale)	2024–2035*
What outcome (Proposed impact of interest)	Improved uptake and coverage of measles-rubella vaccines
**Criteria, alphabetically**	**Description**
Answerability	Do you believe it is possible to design and conduct a study to answer this research question in the populations of interest by 2035?
Equity	Do you believe that the research question and its output(s) will contribute to reducing inequities?
Impact	Do you believe the research question and its output(s) will contribute to increasing coverage of measles-rubella vaccines?
Potential for translation	Do you believe that the research question and its output(s) will contribute to strategies or actions that can be implemented in different country contexts and communities?
Relevance	Do you believe that the research question and its output(s) will be relevant to the defined research context (*e.g.* communities in countries that use measles-rubella and to increase measles-rubella vaccine coverage)?

*Anticipates licensure of measles-rubella microarray patch in 2030 and allows the ability to conduct research and gather learnings after initial introductions.

Table S2. Demographics of survey respondents

Demographics of survey respondents	n (%)
**Geographical region by World Health Organization regions**	
Africa	25 (18)
Americas	41 (29)
Europe	19 (14)
Eastern Mediterranean	8 (6)
South-East Asia	22 (16)
Western Pacific	24 (17)
**Total**	139 (100)
**Geographical location by 2024 World Bank income groups**	
Low income	14 (10)
Lower middle income	41 (29)
Upper middle income	23 (17)
High income	61 (44)
**Total**	139 (100)
**Organisational affiliation**	
Academic institution/research institution	27 (19)
Civil society or non-governmental organisation	30 (22)
Donor	10 (7)
Government agency or department	26 (19)
United Nations agency	39 (28)
Other	7 (5)
**Total**	139 (100)
**Familiarity with measles-rubella microarray patch**	
None at all	14 (10)
A little	22 (16)
A moderate amount	41 (29)
A lot	30 (22)
A great deal	32 (23)
**Total**	139 (100)
**Level of experience implementing health products and interventions in low- and middle-income countries**	
None at all	8 (6)

A little	15 (11)
A moderate amount	33 (24)
A lot	37 (27)
A great deal	46 (33)
**Total**	139 (100)
